# Supplementary material for: Lung Adenocarcinoma Cell Sensitivity to Chemotherapies: A Spotlight on Lipid Droplets and SREBF1 Gene
Source: Cancers (Basel). 2022 Sep 14;14(18):4454. doi: 10.3390/cancers14184454 (PMC9497419; doi:10.3390/cancers14184454)
Supplement: Supplementary file 1 [file cancers-14-04454-s001.zip › cancers-1843057-supplementary.pdf]

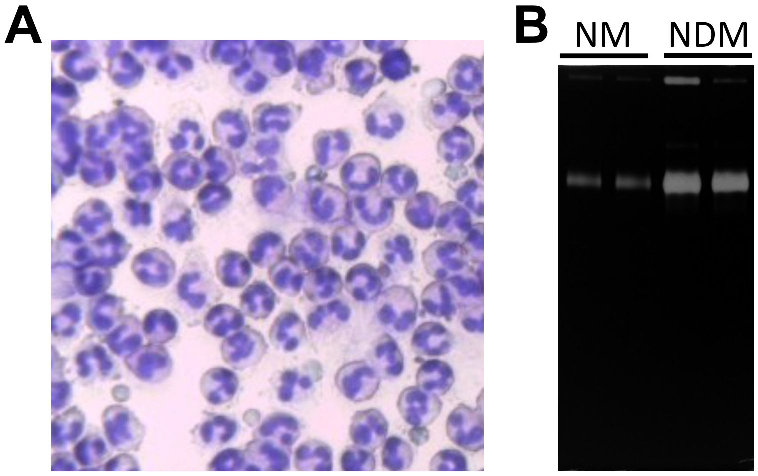

C

MPO (pg/ml)	
NM	NDM
100200	353655
147465	416120
162830	550835

Supplementary Figure S1: Neutrophil isolation and degranulation. A A representative cytospin stained with May-Grünwald and Giemsa. **B** Characterization of neutrophil degranulation by increased protease activity in neutrophil degranulation medium (NDM) as compared to non-degranulated neutrophil medium (NM) on 10% Zymogram gels. One representative gel out of 10 is shown. **C** Increased levels of myeloperoxidase (MPO, pg/ml) in NDM compared to NM as measured by ELISA. Data are shown from three representative experiments out of 30.

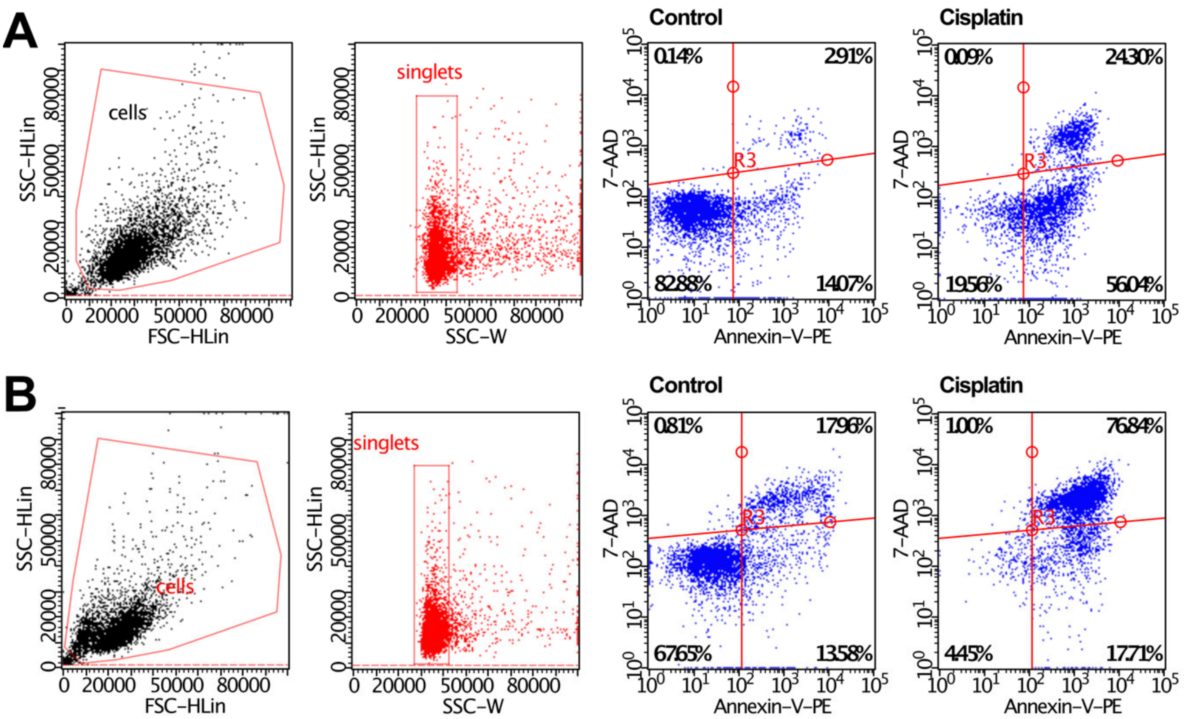

Supplementary Figure S2: Representative analysis of the viability of H1299 (A) and H1563 (B) cells in different media. H1299 and H1563 cells were cultured for 48 h in the presence or absence of 20 µg/ml cisplatin in basal medium. PE Annexin V apoptosis detection assay was used for flow cytometry to measure cell death. Unstained cells in the bottom left quadrant were classified as “living”.

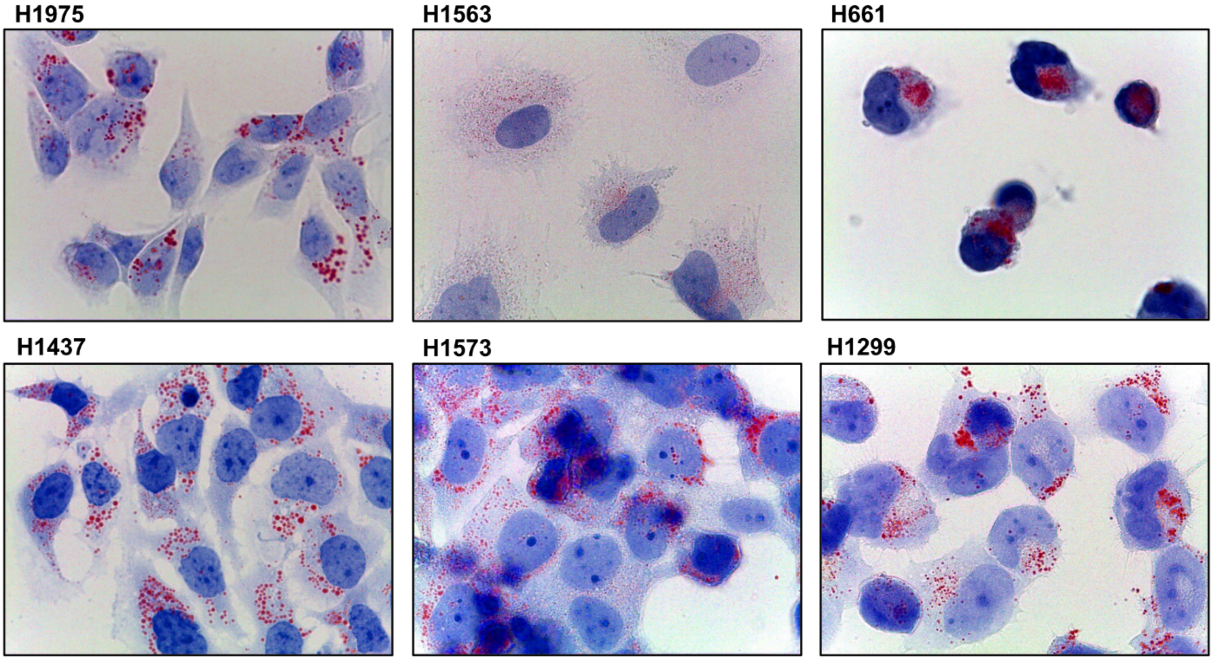

Supplementary Figure S3: Lipid droplet formation in six lung cancer cell lines after treatment with 20µg/ml cisplatin. Cells were stained for lipid droplets with Oil red O and for nuclear components with hematoxylin after culture for 48 h in basal medium. Images were taken at 1000-fold magnification using 100x oil immersion objective (Leica). One representative experiment out of three independent experiments is shown.

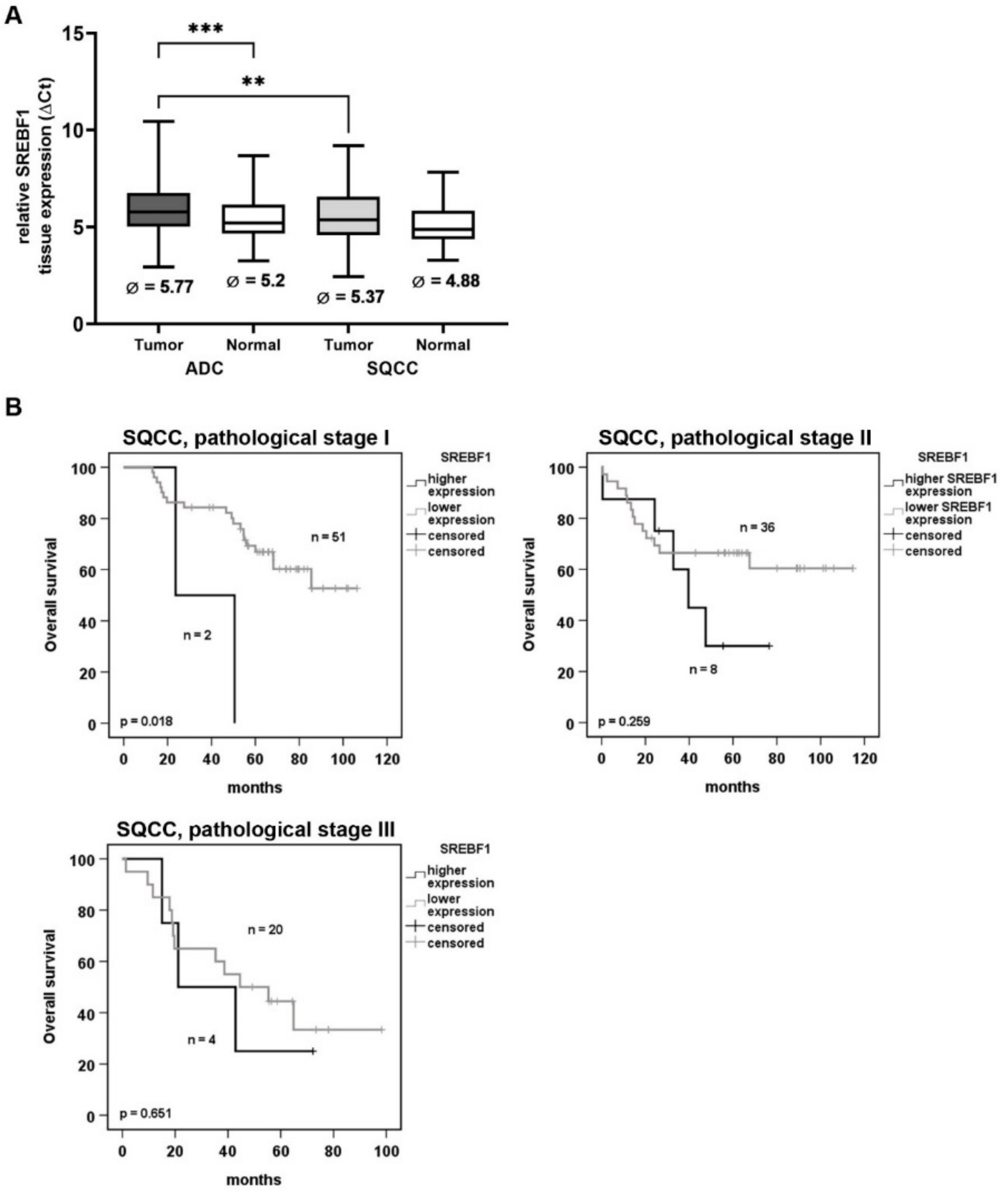

Supplementary Figure S4: *SREBF1* expression in ADC and SQCC and overall survival for SQCC. **A** Relative *SREBF1* expression in tumor and adjacent lung tissue. Gene expression was normalized with two housekeeping genes (*ESD* and *RPS18*). Please note that higher values mean a lower gene expression. **B** Kaplan-Meier survival curves of *SREBF1* tumor expression in SQCC. Cut-off for separation of the groups was the same as used in Fig. 5C. SQCC = squamous cell carcinoma, Ø = median, *p < 0.05, **p < 0.005

Supplementary Table S1. Cohort description		
**Parameter**	**n**	**(%)**
Median Age	65 (38–88)	
**Total**	**292**	**100**
Male	205	70
Female	87	30
**Histology**		
Adeno	171	59
Squamous	121	41
**Therapy**		
OP	179	63
OP/RT	7	2
OP/ChT	76	26
OP/RT/ChT	30	10
**Smoking status**		
Non-Smoker	26	9
Ex-Smoker	162	55
Smoker	102	35
No data	2	1
**Pathological Stage (7th TNM edition)**		
IA	33	11
IB	108	37
IIA	21	7
IIB	62	21
IIIA	63	21
IIIB	5	2
**ECOG**		
0	251	86
1	39	13
2	2	1

OP: operation, RT: radiotherapy, ChT: chemotherapy, ECOG: Eastern Cooperative Oncology Group Performance Status Scale

Supplementary Table S2. Cox Regression Analyzes		
**Univariate Analysis** (Gene Expression, overall survival)		
**Variable (high *vs*. low)**	**Significance**	**Hazard Ratio (95 % CI)**
SREBF1 (ADC)	**0.039**	**1.675 (1.20-2.751)**
SREBF1 (SQCC)	**0.015**	**2.305 (1.15-4.619)**
**Multivariate Analysis (SQCC)** (Gene Expression, Overall Survival)		
**Variable**	**Significance**	**Hazard Ratio (95 % CI)**
SREBF1 (tumor, high vs. low)	**0.012**	**2.571 (1.236-5.348)**
Age	**0.007**	**1.05 (1.014-1.088)**
Gender (female vs. male)	**0.025**	**0.261 (0.08-0.847)**
pathological stage (7th edition)	0.097	1.369 (0.944-1.984)
Smoking status	0.265	1.371 (0.787-2.389)
ECOG	0.569	1.251 (0.579-2.7)
**Multivariate Analysis (ADC)** (Gene Expression, Overall Survival)		
**Variable**	**Significance**	**Hazard Ratio (95 % CI)**
SREBF1 (tumor, high vs. low)	0.527	1.188 (0.697-2.024)
Age	**0.006**	**1.04 (1.011-1.07)**
Gender (female vs. male)	0.18	0.709 (0.429-1.172)
pathological stage (7th edition)	**<0.001**	**1.998 (1.498-2.679)**
Smoking status	**0.047**	**1.262 (1.003-1.587)**
ECOG	0.99	1.004 (0.546-1.846)

ADC: adenocarcinoma, SQCC: squamous cell carcinoma, CI: confidence interval, ECOG: Eastern Cooperative Oncology Group Performance Status Scale
